# Maternal SARS-CoV-2 vaccination and infant protection against SARS-CoV-2 during the first six months of life

**DOI:** 10.1038/s41467-023-36547-4

**Published:** 2023-02-28

**Authors:** Ousseny Zerbo, G. Thomas Ray, Bruce Fireman, Evan Layefsky, Kristin Goddard, Edwin Lewis, Pat Ross, Saad Omer, Mara Greenberg, Nicola P. Klein

**Affiliations:** 1 Kaiser Permanente Northern California, Vaccine Study Center, Oakland, CA USA; 2 Yale University, Institute for Global Health, New Haven, CT USA; 3 Department of Internal Medicine (Infectious Diseases), Yale School of Medicine, New Haven, CT USA; 4 Department of Epidemiology of Microbial Diseases, Yale School of Public Health, New Haven, CT USA; 5 Obstetrics and Gynecology, Kaiser Permanente Northern California Oakland, Oakland, CA USA; 6 Regional Perinatal Service Center, Kaiser Permanente Northern California, Santa Clara, CA USA

**Keywords:** Epidemiology, Paediatric research, Viral infection, Vaccines, SARS-CoV-2

## Abstract

We examined the effectiveness of maternal vaccination against SARS-CoV-2 infection in 30,311 infants born at Kaiser Permanente Northern California from December 15, 2020, to May 31, 2022. Using Cox regression, the effectiveness of ≥2 doses of COVID-19 vaccine received during pregnancy was 84% (95% confidence interval [CI]: 66, 93), 62% (CI: 39, 77) and 56% (CI: 34,71) during months 0–2, 0–4 and 0- 6 of a child’s life, respectively, in the Delta variant period. In the Omicron variant period, the effectiveness of maternal vaccination in these three age intervals was 21% (CI: −21,48), 14% (CI: −9,32) and 13% (CI: −3,26), respectively. Over the entire study period, the incidence of hospitalization for COVID-19 was lower during the first 6 months of life among infants of vaccinated mothers compared with infants of unvaccinated mothers (21/100,000 person-years vs. 100/100,000 person-years). Maternal vaccination was protective, but protection was lower during Omicron than during Delta. Protection during both periods decreased as infants aged.

## Abstract (web summary)

This study investigates the impact of maternal COVID-19 vaccination during pregnancy on infant infection during the first six months of life. Using data from California, USA, the authors find that protection against infection during the period of Delta dominance was high, but that it declined during the Omicron period.

## Introduction

In the US, as of the end of September 2022, almost 15 million children ages <18 years have tested positive for severe acute respiratory syndrome coronavirus 2 (SARS-CoV-2), the virus that causes Coronavirus Disease 2019 (COVID-19). Children currently account for about 18.5% of reported COVID-19 cases in the United States^1^. SARS-CoV-2 infection can lead to severe illnesses and hospitalizations in children and infants^2–5^. During Omicron predominance, children aged <6 months accounted for 44% of hospitalizations among children ages 0–4 years^3^.

Vaccination offers the best way to protect against COVID-19 and its complications. COVID-19 vaccines have demonstrated both high efficacy in clinical trials and high real-world effectiveness, especially against the original and Delta variant of the virus^6–10^. Real-world data suggest lower COVID-19 vaccine effectiveness against Omicron variants^11–14^. However, infants aged <6 months are not currently eligible for any currently available COVID-19 vaccines and must rely on placentally acquired immunity from their mothers.

Like influenza and Tdap vaccines^15,16^, data suggest that vaccination during pregnancy may protect infants who are not old enough to be vaccinated against COVID-19. Three recent epidemiological studies found that vaccination during pregnancy was associated with a reduced risk of SARS-CoV-2 infection in infants during their first 4 months of life and a reduced risk of hospitalization during the first 5 months of life^17–19^.

The objective of this study was to further evaluate the effectiveness of at least two doses of mRNA COVID-19 vaccination during pregnancy for preventing SARS-CoV-2 infection in infants during the first 2, 4, and 6 months of life during the Delta and Omicron variant periods. We used two different study designs: a primary design using a cohort analysis in which infants of vaccinated pregnant persons were compared with infants of unvaccinated pregnant persons. In this design, we used Cox proportional hazards models with calendar days as the underlying scale to estimate hazard ratios and calculated vaccine effectiveness as 1 minus the hazard ratio. Secondarily, we used a Test-Negative Design (TND), which is a case-control study, to compare the odds of vaccination among mothers of infants who tested positive vs. the odds of vaccination among mothers of infants who tested negative. In this analysis, vaccine effectiveness was evaluated as 1 minus the odds ratio. The aim of the secondary design was to compare the results of the cohort with TND.

## Results

### Descriptive statistics and characteristics

**Fig. 1:**
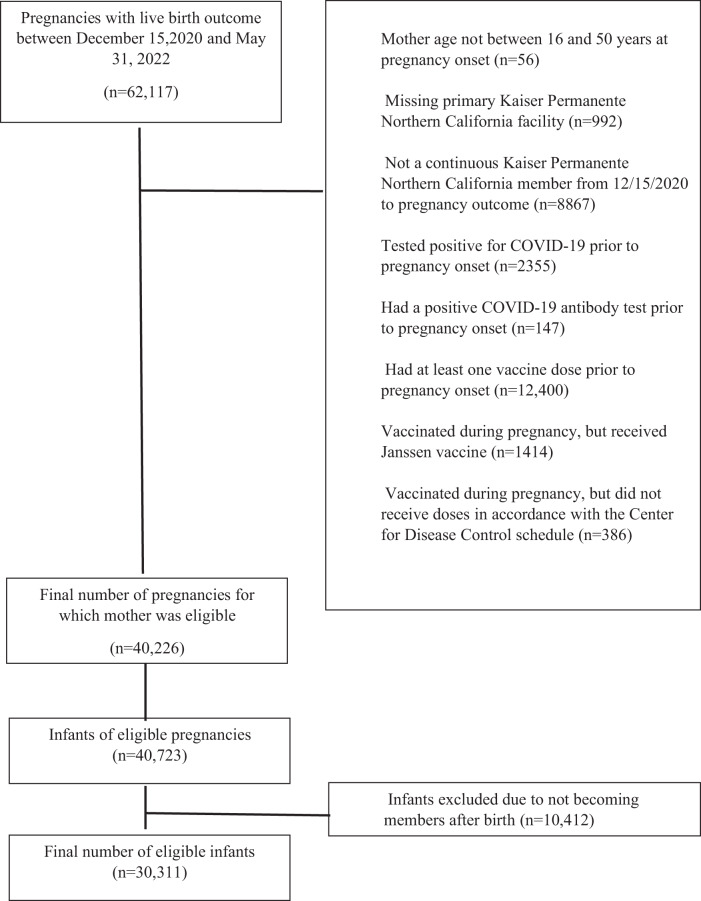
Construction of study cohort. Children born at Kaiser Permanente Northern California from December 15, 2020, through May 31, 2022.

Between December 15, 2020, and May 31, 2022, we identified 62,117 infants born at Kaiser Permanente Northern California (KPNC), an integrated healthcare delivery organization. Among these infants, for our main analysis, we excluded 21,891 (35.2%) based on maternal exclusion criteria and 10,412 (16.8%) after applying infant exclusion criteria (Fig. 1). The final study population included 30311 (48.8%) infants who were KPNC members at least 2 months after birth. The mean age at pregnancy onset was 31.62 years (standard deviation of 4.66 years). Most mothers (66.14%) were between ages 25 and <35 years, and more than a quarter (27.27%) were of Asian race, 5.16% were Black, 24.44% were of Hispanic ethnicity and 37.57% were White. Among the infants in the cohort, 19,418 (64.06%) of the mothers were unvaccinated during pregnancy, 1138 (3.75%) of the mothers received one dose of an mRNA COVID-19 vaccine and 9755 (32.18%) received ≥2 doses during pregnancy (Table 1). Most mothers (1032 of 1138) who received only one dose received the vaccine during the third trimester.

**Table 1:** Characteristics of the study population: infants born at Kaiser Permanente Northern California December 15, 2020—May 31, 2022

Characteristics	Infants included in the study *N* = 30,311 *n* (%)	Infants whose mothers were vaccinated during pregnancy *N* = 10,893 *n*(%)	Infants whose mothers were not vaccinated during pregnancy *N* = 19,418 *n* (%)
Mother’s age at pregnancy onset (years), mean (std)	31.62 (4.66)	32.59 (4.30)	31.08 (4.77)
**Mother’s age category (years)**			
16–<25	2092 (6.90)	384 (3.53)	1708 (8.80)
25–<35	20,049 (66.14)	6915 (63.48)	13,134 (67.64)
35–<50	8170 (26.95)	3594 (32.99)	4576 (23.57)
**Mother’s race/ethnicity**			
Asian	8266 (27.27)	3682 (33.80)	4584 (23.61)
Black	1564 (5.16)	365 (3.35)	1199 (6.17)
Hispanic	7408 (24.44)	2146 (19.70)	5262 (27.10)
Pacific Islander	246 (0.81)	72 (0.66)	174 (0.90)
Multiracial	108 (0.36)	36 (0.33)	72 (0.37)
Native American	765 (2.52)	288 (2.64)	477 (2.46)
Other/unknown/multiracial	567 (1.87)	218 (2.00)	349 (1.80)
White	11,387 (37.57)	4086(37.51)	7301 (37.60)
**Parity**			
0	12,091 (39.89)	4716 (43.29)	7375 (37.98)
1	11,189 (36.91)	4176 (38.34)	7013 (36.12)
2	3925 (12.95)	1144 (10.50)	2781 (14.32)
3	1213 (4.00)	274 (2.52)	939 (4.84)
4+	576 (1.90)	129 (1.18)	447 (2.30)
Unknown	1317 (4.34)	454 (4.17)	863 (4.44)
**Medical comorbidity before pregnancy**			
Diabetes	491 (1.62)	217 (1.99)	274 (1.41)
Hypertension	2469 (8.15)	889 (8.16)	1580 (8.14)
**Body mass index**			
Underweight	659 (2.17)	266 (2.44)	393 (2.02)
Normal	12,488 (41.20)	4773 (43.82)	7715 (39.73)
Overweight	8845 (29.18)	3066 (28.15)	5779 (29.76)
Obese	8212 (27.09)	2753 (25.27)	5459 (28.11)
Unknown	107 (0.35)	35 (0.32)	72 (0.37)
**Type of insurance**			
Subsidized (Medicare/Medicaid/other subsidized insurance)	2008 (6.62)	445 (4.09)	1563 (8.05)
Non-subsidized	28,303 (93.38)	10,448 (95.91)	17,855 (91.95)
**Neighborhood deprivation index (quartile)**			
First quartile 1 (least deprived)	7485 (24.69)	3584 (32.90)	3901 (20.09)
Second quartile	8112 (26.76)	2953 (27.11)	5159 (26.57)
Third quartile	7261 (23.95)	2328 (21.37)	4933 (25.40)
Fourth quartile (most deprived)	7400 (24.41)	2006 (18.42)	5394 (27.78)
Missing	53 (0.17)	22 (0.20)	31 (0.16)
**mRNA COVID-19 vaccine doses**			
0 dose	19,418 (64.06)	0 (0.00)	19,418 (100)
1 dose	1138 (3.75)	1138 (10.45)	0 (0.00)
2+ doses	9755 (32.18)	9755 (89.55)	0 (0.00)
**Gestational age at receipt of first COVID-19 vaccine dose among women receiving only one dose**			
First trimester	60 (0.20)	60 (0.55)	0 (0.00)
Second trimester	46 (0.15)	46 (0.42)	0 (0.00)
Third trimester	1032 (3.40)	1032 (9.47)	0 (0.00)
**Gestational age at receipt of second COVID-19 vaccine dose among women receiving 2+ doses**			
First trimester	2082 (6.87)	2082 (19.11)	0 (0.00)
Second trimester	3671 (12.11)	3671 (33.70)	0 (0.00)
Third trimester	4002 (13.20)	4002 (36.74)	0 (0.00)
**PCR status**			
Positive during first 6 months of life	940 (3.10)	385 (3.53)	555 (2.86)
**Hospitalization status**			
Hospitalized with positive PCR during first 6 months of life	10 (0.03)	1 (<0.01)	9 (0.05)

During the first 6 months of life, 940 (3.10%) infants tested positive for SARS-CoV-2 by polymerase chain reaction (PCR) test and 10 (0.03%) infants were hospitalized with a positive SARS-CoV-2 test.

### Vaccine effectiveness: primary design cohort analyses

During the Delta dominant period, the crude incidences of testing positive for SARS-CoV-2 during the first 2, 4, and 6 months of life were lower among infants whose mothers received at least two doses of mRNA COVID-19 vaccines during pregnancy (0.75, 1.43, and 1.56 infants per 100 person-years [PY], respectively) than those whose mothers were not vaccinated during pregnancy (5.47, 5.10, and 4.78 infants per 100 PYs, respectively). After adjusting for covariates, vaccination during pregnancy significantly reduced the risk of the infant testing SARS-CoV-2 positive by 84% (95% confidence interval [CI]: 66, 93) during the first 2 months of life, 62% (95% CI: 39, 77) during the first 4 months of life and 56% (95% CI: 34,71) during the first 6 months of life. Vaccine effectiveness for 1 dose during the first 6 months of life was 68% (95% CI: 12, 88) (Table 2). During the Omicron dominant period, receipt of ≥2 doses during pregnancy reduced the risk of the infant testing SARS-CoV-2 positive by 21% (95% CI: −21, 48) during the first 2 months of life, 14% (95% CI: −8, 32) during the first 4 months of life, and 13% (95% CI: − 3, 26) during the first 6 months of life (Table 2). All these results were similar to those when no adjustments for covariates are made (Supplemental Table 1).

**Table 2:** Effectiveness of COVID-19 vaccination during pregnancy against infant SARS-CoV-2 infection: cohort design

	First 2 months of life			First 4 months of life			First 6 months of life		
Infant observation period by predominant SARS-CoV-2 variant, and mother’s vaccination status	Positive test *N*	Crude incidence rate^a^	Adjusted^b^ VE (95% CI)	Positive test *N*	Crude incidence rate^a^	Adjusted^b^ VE (95% CI)	Positive test *N*	Crude incidence rate^a^	Adjusted^b^ VE (95% CI)
**Delta period**									
Unvaccinated during pregnancy	54	5.47	Reference	118	5.10	Reference	190	4.78	Reference
Received ≥2 doses during pregnancy	8	0.75	84 (66,93)	27	1.43	62 (39,77)	37	1.56	56 (34,71)
Received only 1 dose during pregnancy^c^	0	0.00	no estimate	0	0.00	no estimate	4	1.33	68 (12,88)
**Omicron period**									
Unvaccinated during pregnancy	54	16.98	Reference	140	18.32	Reference	302	21.11	Reference
Received ≥2 doses during pregnancy	54	16.30	21 (−21,48)	175	17.25	14 (−8,32)	320	16.34	13 (−3,26)
Received only 1 dose during pregnancy^c^	1	7.87	50 (−72,93)	8	17.79	36 (−23,68)	23	22.73	7 (−29,39)

Calendar day was used as the underlying scale to ensure that infants of vaccinated and unvaccinated mothers are compared on the same calendar days.

*VE* vaccine effectiveness.

^a^Rate per 100 person-years.

^b^Adjusted for mother’s age, race/ethnicity, neighborhood deprivation index quartile, insurance payor, KPNC facility, pre-pregnancy BMI, diabetes, hypertension, parity, and child age.

^c^Most one dose only vaccines were received during the third trimester.

In supplemental analyses by trimester of vaccination, receipt of the second dose during the second and third trimesters reduced the risk of infants testing SARS-CoV-2 positive during the Delta dominant period by 91% (95% CI: 63, 98) and 85% (95% CI: 50, 96), respectively, during the first 2 months of life, by 59% (95% CI: 21, 79) and 67% (95% CI: 37, 83) during the first 4 months of life and by 64% (95% CI:31, 81) and 53% (95% CI: 24, 71) during the first 6 months of life. During the Delta period, receipt of one dose during the third trimester reduced infants’ risk of testing positive for SARS-CoV-2 by 74% (95% CI: 19, 92) during the first 6 months of life (Table 3). We observed a similar pattern in vaccine effectiveness by trimester during the Omicron dominant period, however, estimates of vaccine effectiveness by trimester were imprecise and much lower than during the Delta period (Table 3).

**Table 3:** Effectiveness of COVID-19 vaccination during pregnancy against infant SARS-CoV-2 infection by infant age at testing, trimester of vaccination during pregnancy, and by virus variant: cohort design

	First 2 months of life			First 4 months of life			First 6 months of life		
Infant observation period by predominant SARS-CoV-2 variant, and mother’s vaccination status	Positive test *N*	Crude incidence rate^a^	Adjusted^b^ VE (95% CI)	Positive test *N*	Crude incidence rate^a^	Adjusted^b^ VE (95% CI)	Positive test *N*	Crude incidence rate^a^	Adjusted^b^ VE (95% CI)
**Delta period**									
Unvaccinated during pregnancy	54	5.47	Reference	118	5.10	Reference	190	4.78	Reference
**Received ≥2 doses during pregnancy**									
Second dose during the first trimester	3	1.73	62 (−23,89)	4	1.80	46 (−36, 82)	4	1.78	47 (−34, 81)
Second dose during the second trimester	2	0.42	91 (63,98)	11	1.39	59 (21,79)	11	1.23	64 (31,81)
Second dose during the third trimester	3	0.72	85 (50,96)	12	1.37	67 (37,83)	22	1.77	53 (24,71)
**Received only one dose during pregnancy**									
First dose during the first trimester	0	0.00	no estimate	0	0.00	no estimate	0	0.00	no estimate
First dose during the second trimester	0	0.00	no estimate	0	0.00	no estimate	1	9.30	−58 (−94, 64)
First dose during the third trimester	0	0.00	no estimate	0	0.00	no estimate	3	1.06	74 (19, 92)
Omicron period									
Unvaccinated during pregnancy	54	16.98	Reference	140	18.32	Reference	302	21.11	Reference
**Received ≥2 doses during pregnancy**									
Second dose during the first trimester	26	15.67	25 (−19,55)	58	13.18	24 (−5,45)	86	12.23	20 (−2, 38)
Second dose during the second trimester	12	11.26	36 (−18, 67)	72	19.74	6 (−21, 30)	152	19.16	4 (−16, 22)
Second dose during the third trimester	16	27.22	−8 (−48, 38)	45	21.50	12 (−19, 37)	82	17.75	18 (−4, 36)
**Received only one dose during pregnancy**									
First dose during the first trimester	0	0.00	no estimate	0	0.00	no estimate	1	6.16	66 (−57, 95)
First dose during the second trimester	0	0.00	no estimate	0	0.00	no estimate	3	35.14	−44 (−82, 43)
First dose during the third trimester	1	16.41	3 (−86, 87)	8	26.32	19 (−39, 60)	19	24.86	6 (−33, 40)

Calendar day was used as the underlying scale to ensure that infants of vaccinated and unvaccinated mothers are compared on the same calendar days.

VE vaccine effectiveness.

^a^Rate per 100 person-years.

^b^Adjusted for mother’s age, race/ethnicity, neighborhood deprivation index quartile, insurance payor, KPNC facility, pre-pregnancy BMI, diabetes, hypertension, parity, and child age.

Over the entire study period, the crude rate of hospitalization with a SARS-CoV-2 positive test was lower during the first 6 months of life among infants whose mothers received at least two doses of mRNA COVID-19 vaccines during pregnancy compared with infants whose mothers were unvaccinated during pregnancy (21/100,000 PY vs. 100/100,000 PY). VE against hospitalization was not estimated because of the very small number of hospitalized cases. There were only one hospitalized case among the children of vaccinated mothers and nine hospitalized cases among the children of unvaccinated mothers (Table 1).

### Secondary analysis results using a test-negative design (TND)

In the TND, we estimated that during the Delta predominant period, maternal vaccination with at least doses reduced the infant’s risk of testing SARS-CoV-2 positive by 95% (95% CI:76, 99) during the first 2 months of life, 70% (95% CI: 52, 82) during the first 4 months of life, and 61% (95% CI: 42, 74) during the first 6 months of life (Supplemental Table 2). During the Omicron dominant period, maternal vaccination with at least two doses reduced the infant’s risk of testing SARS-CoV-2 positive by 43% (95% CI: −4, 69) during the first 2 months of life, 36% (95% CI:11, 55) during the first 4 months of life, and 41% (95% CI: 25, 53) during the first 6 months of life (Supplemental Table 2). The results were unchanged when no adjustments for covariates were made (Supplemental Table 3).

### Additional supplemental analysis

During the Omicron period, among children whose mothers received 1 dose before pregnancy and 1 dose during pregnancy, VE against infection was 46% (95% CI: −23, 77) during the first 2 months of life, 16% (95% CI: −28, 50) during the first 4 months of life and 3% (95% CI: −32, 36) during the first 6 months of life compared with children whose mothers were unvaccinated (Supplemental Table 4). For children whose mothers received one dose before pregnancy and two doses during pregnancy, VE against infection was 89% during the first 2 months of life, 73% during the first 4 months of life, and 48% up to 6 months of life.

## Discussion

In this large study which included >30,000 infants, we found that receipt of at least two doses of mRNA COVID-19 vaccine during pregnancy was associated with a decreased risk of infants testing SARS-CoV-2 positive during their first 6 months of life. Maternal vaccination with at least two doses reduced the infant’s risk of testing SARS-CoV-2 positive initially by 84% which decreased to 56% by 6 months of life in the Delta dominant period. Receipt of one dose especially during the third trimester was also associated with a reduced risk of infants testing positive for SARS-CoV-2 during the first 6 months of life during the Delta dominant period. However, vaccination during pregnancy was less effective at protecting infants against SARS-CoV-2 infection during the Omicron period. As infants aged, protection provided by maternal vaccination decreased during both periods.

Although the study was unable to directly estimate VE against hospitalization due to the small number of hospitalized cases, it found that over the entire study period, the incidence rate of hospitalization during the first 6 months of life was much lower among the infants whose mothers were vaccinated during pregnancy compared with those whose mothers were not vaccinated. These results suggest that in addition to providing protection against testing positive, vaccination during pregnancy also provides protection against hospitalization (severe disease) in infants during their first 6 months of life as previously reported recently^18,19^.

Our findings that receipt of at least two doses of COVID-19 vaccine during pregnancy was effective at protecting infants during the Delta period are similar to those reported in a recent Norwegian study showing that mRNA COVID-19 vaccination during pregnancy was associated with a 71% decreased risk of testing positive for SARS-CoV-2 in infants during their first 4 months of life during the Delta period^17^. During the Delta period, we found that protection extended through the infant’s first 6 months of life. However, in contrast with the Norwegian study which reported that infants of mothers who were vaccinated had a 33% decreased risk of testing positive during the first 4 months of life during the Omicron period^17^, our study found a 13% reduced risk that was not statistically significant. The difference between the two studies might be due to population characteristics and the timing of follow-up as ours went through May 31, 2022, while the Norwegian study ended in April 2022.

The finding that maternal vaccination was less effective at protecting infants during the Omicron dominant period is also consistent with previous studies which have reported decreased effectiveness of mRNA COVID-19 vaccines during Omicron among children and adults^14,20^. Recently another study reported that the effectiveness of mRNA COVID-19 vaccines against infections and hospitalizations among pregnant people was higher during the Delta period than during the Omicron period^21^.

Our additional supplemental analysis suggests that pregnant persons who received at least one vaccine dose before pregnancy should complete their vaccination series during pregnancy to provide protection to their children during the first 6 months of life.

We observed that infants’ protection through vaccination during pregnancy decreased as they aged from 2 months to 6 months. These findings are consistent with the diminishing of pregnancy-derived antibodies in infants over time^22^. A recent study found that the mean titer of maternally derived antibodies in infants of vaccinated mothers were higher at age 2 months compared with antibody titers at age 6 months^23^.

Despite several studies showing that vaccination during pregnancy is safe for pregnant people^24–28^, vaccine uptake has been suboptimal in this group^29^. In the present study, the mothers of only 32% of infants in the cohort received at least 2 doses during pregnancy. While this proportion might not be representative of the proportion of vaccinated pregnant women within KPNC because of our exclusion criteria, more efforts are needed to promote COVID-19 vaccines for pregnant persons because vaccination provides protection to mothers and their infants until they are old enough to receive their own COVID-19 vaccines.

Our study was strengthened both by its large sample size and our ability to follow infants through 6 months of age. In addition, our study period included two different SARV-CoV-2 variants, which allowed estimation of the effectiveness of vaccination during pregnancy in infants during both the Delta and Omicron variant periods. Our primary cohort analysis used calendar days as the underlying scale to ensure that we compared infants of vaccinated and unvaccinated mothers on the same calendar days because vaccination status during pregnancy and risk of SARS-CoV-2 infection varied over the study period. In this primary design, all eligible infants meeting inclusion criteria were included without sampling which improved power and minimized bias related to selection. Furthermore, it was reassuring that both the cohort and the secondary TND yielded vaccine effectiveness estimates in the same direction. Although both approaches adjusted for the same confounding factors, the effectiveness estimates from the TND were higher than those from the cohort design, which is consistent with our previous analyses of influenza vaccine effectiveness in which we also observed that the TND tended to result in higher vaccine effectiveness estimates than did our cohort analyses^30^. The TND, a case-control study, has been commonly used in studies of the effectiveness of influenza vaccines and more recently COVID-19 vaccines. The cohort analyses may be biased toward the null if some infected infants were misclassified as uninfected due to the absence of a SARS-CoV-2 test result in the medical record. The TND analyses avoid this bias by limiting the analysis to infants who were tested for SARS-CoV-2. Thus, this design better adjusts for healthcare-seeking behavior^31,32^, but it may also introduce other biases including selection bias^33^.

The study had limitations worth noting. Vaccinations were limited only to those received during pregnancy. We did not assess whether vaccines received before pregnancy or immediately after pregnancy were associated with a reduced risk of testing positive for SARS-CoV-2 in infants. The study did not adjust for maternal SARS-CoV-2 infections during pregnancy due to the inability of capturing home testing results. We, therefore, were unable to assess whether maternal infection provided some protection to their infants. Additional limitations include the  inability to estimate the effectiveness of vaccines received prior to pregnancy onset. It is possible that our exclusion criteria may have resulted in a final sample that may not be reflective of all KPNC infants. During the study period, home testing became more prevalent. It is possible that this practice may have led to some misclassification of the outcome, and we were unable to assess whether this misclassification was differential between vaccinated and unvaccinated mothers. We did not have genotyping data to confirm the variant that infected infants who tested positive and instead relied on state data regarding circulating strain predominance in the Northern California region. Like all observational studies, our study results are susceptible to residual confounding.

In conclusion, in this population-based cohort study, we found that infants born to mothers who received at least two doses of an mRNA COVID-19 vaccine during pregnancy were at lower risk of testing positive for SARS-CoV-2 and were at lower risk of hospitalization during the first 6 months of life compared with infants whose mothers were unvaccinated during pregnancy. Maternal vaccination was protective, but protection was lower during the Omicron period than during Delta. Protection during both periods decreased as infants aged from 2 months to 6 months. Overall, the study results support recommendations for vaccination during pregnancy to protect both mothers and their infants.

## Methods

### Setting and study population

The study setting was Kaiser Permanente Northern California (KPNC), an integrated healthcare delivery organization that provides comprehensive healthcare to ~4.4 million members as of 2019. Members receive almost all their medical care at KPNC-owned facilities, including clinics, hospitals, pharmacies, and laboratories. KPNC has a comprehensive electronic health record system (Kaiser Permanente HealthConnect®, a customized EPIC system), that captures detailed information on all medical services, including immunization, membership enrollment including place of residence, demographics, and pregnancy-related care from pregnancy onset to delivery, and beyond. KPNC members are similar to the broad catchment population in Northern California in terms of sociodemographic characteristics^34^. Annually, approximately 40,000 births occur at KPNC facilities.

The study was conducted among a cohort of infants born between December 15, 2020, and May 31, 2022. From this cohort, the study excluded the following infants born to (1) mothers who were not between ages 16 and 50 years at pregnancy onset; (2) mothers who did not have a primary KPNC facility assignment; (3) mothers who were not continuous KPNC members from December 15, 2020 until delivery; (4) mothers who had a positive nasal/throat swab for SARS-CoV-2 by polymerase chain reaction (PCR) prior to pregnancy onset; (5) mothers who had a positive SARS-CoV-2 antibody test documented by KPNC prior to the onset of pregnancy; (6) mothers who received one or more doses of COVID-19 vaccine prior to pregnancy onset. We excluded these infants because we were primarily interested in estimating the effectiveness of mRNA vaccines received during pregnancy; (7) mothers who received adenovirus vector vaccines or any non-mRNA platform vaccines during pregnancy; (8) mothers who did not receive their mRNA vaccinations in accordance with CDC recommendations—e.g., the timing between dose 1 and dose 2 was not within the recommended intervals; and (9) infants who did not become KPNC members within two calendar months of their birth. No other exclusion criteria were applied.

The KPNC Institutional review board approved and waived consent for this study. Informed consent was waived because this was a data-only study with no direct contact with participants.

### Outcomes

The outcomes were the infant’s first positive nasal/throat swab for SARS-CoV-2 by PCR, and the first COVID-19-related hospitalization, occurring during the first 6 months of life and recorded in the electronic health record.

### Exposure

The exposure of interest was mRNA COVID-19 vaccination status during pregnancy in the electronic health record. Mothers were classified as either having had ≥2 doses or one dose of mRNA COVID-19 vaccines during pregnancy (and completed more than 14 days prior to delivery) or not having had any COVID-19 vaccines prior to delivery. We further classified vaccination status by the trimester within which the second dose or the unique dose (for those who received only one dose) was received.

### Covariates

For mothers of infants in the cohort, we extracted from the electronic health record data: age at pregnancy onset, self-reported race/ethnicity (Asian, Black, Hispanic, Pacific Islander, Multiracial, Native American, Other, White), the primary KPNC facility at which the woman received most of their health care, insurance payor (dichotomized as “Medicare/Medicaid/other subsidized insurance” and “Other”), neighborhood deprivation index [NDI]^35^ categorized into quartiles with higher values representing greater deprivation), pre-pregnancy body mass index (BMI = kg/m^2^; underweight <18.5, normal 18.5–24.9, overweight 25.0–29.9, obese ≥30.0), pre-pregnancy diabetes status, pre-pregnancy hypertension, and parity (0, 1, 2, 3, ≥4). For infants, we included age, as a categorical time-changing variable in 30-day increments. All adjustment variables were selected a priori based on prior work^36^.

### Statistical analysis

We conducted a descriptive analysis of the study population and calculated crude rates of SARS-CoV-2 infection and hospitalization by maternal vaccination status. In our primary analysis, we implemented a cohort study design where we used Cox proportional hazards models that allow for time-varying covariates to estimate the SARS-CoV-2 infection hazard ratio (HR) in infants of mothers vaccinated with at least 2 doses of mRNA COVID-19 vaccines during pregnancy and 1 dose only versus mothers who were unvaccinated during pregnancy. We calculated vaccine effectiveness (VE) as 100% multiplied by 1—HR. In all models, we used calendar days as the time scale to account for changes over time in SARS-CoV-2 circulation and vaccine uptake. Infants were followed from birth until the first positive SARS-CoV-2 test by PCR at age 2, 4, or 6 months, with censoring due to death, health plan disenrollment, or end of follow-up (May 31, 2022). Models were adjusted for the covariates listed above. To account for the correlation between infants with the same mother, we fit marginal Cox proportional hazards models using robust sandwich variance estimates. We ran separate models on the time periods associated with the Delta (7/01/2021 to 12/20/2021) and Omicron variants (12/21/2021 to 5/31/2022). We also conducted analyses based on the trimester during which the vaccine was received during pregnancy (first, second, or third trimester).

We conducted secondary sensitivity analyses restricting the population to infants who received at least one SARS-CoV-2 PCR test. In this analysis, we estimated the odds ratio (OR) of vaccination of mothers of infants who tested positive for SARS-CoV-2 versus infants who tested negative using logistic regression models conditioned (stratified) on the calendar date of the test so that infants testing positive on a certain day were compared to infants testing negative on that same day. We calculated VE as 100% multiplied by 1- OR. This case-positive, control-test-negative design also referred to as the test-negative design (TND) has often been used in studies of vaccine effectiveness. The TND is designed to better control for bias related to health care-seeking behavior^31,32^. Models in this analysis were adjusted for the same covariates included in the primary analysis.

Finally, we conducted additional supplemental analyses to estimate VE among children whose mothers received at least one vaccine dose prior to pregnancy onset and at least one dose during pregnancy. All analyses were conducted using SAS software, v9.4. and statistical significance was assessed at two-sided *p* ≤ 0.05.

### Reporting summary

Further information on research design is available in the Nature Portfolio Reporting Summary linked to this article.

## Supplementary information

Supplementary Information

Reporting Summary

## Data Availability

The data cannot be shared publicly because the data contain potentially identifying or sensitive patient information and is legally restricted by Kaiser Permanente Northern California. Data were available for researchers who meet the criteria for access to Kaiser Permanente Northern California confidential data. Data requests may be sent to Kaiser Permanente Division of Research: DOR.IRB.Submissions@kp.org.

## Code availability

Statistical codes are not publicly available but are available from the corresponding author.

## References

[1] American Academy of Pediatrics. Children and COVID-19: state data report. https://downloads.aap.org/AAP/PDF/AAP%20and%20CHA%20-%20Children%20and%20COVID-19%20State%20Data%20Report%209.29.22%20FINAL.pdf?_ga=2.255000394.335550773.1665001859-370326403.1636740765 (2022).

[2] Graff K (2021). Risk factors for severe COVID-19 in children. Pediatr. Infect. Dis. J..

[3] Marks KJ (2022). Hospitalization of infants and children aged 0-4 years with laboratory-confirmed COVID-19—COVID-NET, 14 states, March 2020-February 2022. Morb. Mortal. Wkly. Rep..

[4] Hobbs CV (2022). Frequency, characteristics and complications of COVID-19 in hospitalized infants. Pediatr. Infect. Dis. J..

[5] Kim L (2020). Hospitalization rates and characteristics of children aged <18 years hospitalized with laboratory-confirmed COVID-19—COVID-NET, 14 States, March 1-July 25, 2020. Morb. Mortal. Wkly. Rep..

[6] Baden LR (2021). Efficacy and safety of the mRNA-1273 SARS-CoV-2 vaccine. N. Engl. J. Med..

[7] Polack FP (2020). Safety and efficacy of the BNT162b2 mRNA covid-19 vaccine. N. Engl. J. Med..

[8] Sadoff J (2021). Safety and efficacy of single-dose Ad26.COV2.S vaccine against Covid-19. N. Engl. J. Med..

[9] Dagan N (2021). BNT162b2 mRNA Covid-19 vaccine in a nationwide mass vaccination setting. N. Engl. J. Med..

[10] Thompson MG (2021). Effectiveness of Covid-19 vaccines in ambulatory and inpatient care settings. N. Engl. J. Med..

[11] Buchan SA (2022). Estimated effectiveness of COVID-19 vaccines against omicron or delta symptomatic infection and severe outcomes. JAMA Netw. Open.

[12] Cohen-Stavi CJ (2022). BNT162b2 vaccine effectiveness against omicron in children 5 to 11 years of age. N. Engl. J. Med..

[13] Ferdinands JM (2022). Waning 2-dose and 3-dose effectiveness of mRNA vaccines against COVID-19-associated emergency department and urgent care encounters and hospitalizations among adults during periods of delta and omicron variant predominance—VISION Network, 10 states, August 2021-January 2022. Morb. Mortal. Wkly. Rep..

[14] Klein NP (2022). Effectiveness of COVID-19 Pfizer-BioNTech BNT162b2 mRNA vaccination in preventing COVID-19-associated emergency department and urgent care encounters and hospitalizations among nonimmunocompromised children and adolescents aged 5-17 years—VISION Network, 10 states, April 2021-January 2022. Morb. Mortal. Wkly Rep..

[15] Baxter, R., Bartlett. J., Fireman. B., Lewis. E. & Klein, N. P. Effectiveness of vaccination during pregnancy to prevent infant pertussis. *Pediatrics*. **139**, e20164091 (2017). 10.1542/peds.2016-4091 28557752

[16] Foo D, Sarna M, Pereira G, Moore HC, Regan AK (2022). Longitudinal, population-based cohort study of prenatal influenza vaccination and influenza infection in childhood. Vaccine.

[17] Carlsen EO (2022). Association of COVID-19 vaccination during pregnancy with incidence of SARS-CoV-2 infection in infants. JAMA Intern. Med..

[18] Halasa NB (2022). Maternal vaccination and risk of hospitalization for Covid-19 among infants. N. Engl. J. Med..

[19] Danino D (2022). Effectiveness of BNT162b2 vaccination during pregnancy in preventing hospitalization for SARS-CoV-2 in infants. J. Pediatr..

[20] Andrews N (2022). Covid-19 vaccine effectiveness against the Omicron (B.1.1.529) variant. N. Engl. J. Med..

[21] Schrag SJ (2022). Estimation of COVID-19 mRNA vaccine effectiveness against medically attended COVID-19 in pregnancy during periods of delta and Omicron variant predominance in the United States. JAMA Netw. Open.

[22] Voysey M, Pollard AJ, Sadarangani M, Fanshawe TR (2017). Prevalence and decay of maternal pneumococcal and meningococcal antibodies: a meta-analysis of type-specific decay rates. Vaccine.

[23] Shook LL (2022). Durability of anti-spike antibodies in infants after maternal COVID-19 vaccination or natural infection. JAMA.

[24] Blakeway H (2022). COVID-19 vaccination during pregnancy: coverage and safety. Am. J. Obstet. Gynecol..

[25] DeSilva M (2022). Evaluation of acute adverse events after Covid-19 vaccination during pregnancy. N. Engl. J. Med..

[26] Kharbanda EO (2021). Spontaneous abortion following COVID-19 vaccination during pregnancy. JAMA.

[27] Lipkind HS (2022). Receipt of COVID-19 vaccine during pregnancy and preterm or small-for-gestational-age at birth—eight integrated Health Care Organizations, United States, December 15, 2020-July 22, 2021. Morb. Mortal. Wkly. Rep..

[28] Shimabukuro TT (2021). Preliminary findings of mRNA Covid-19 vaccine safety in pregnant persons. N. Engl. J. Med..

[29] Razzaghi H (2021). COVID-19 vaccination coverage among pregnant women during pregnancy—Eight Integrated Health Care Organizations, United States, December 14, 2020-May 8, 2021. Morb. Mortal. Wkly. Rep..

[30] Klein NP (2020). Vaccine effectiveness of cell-culture relative to egg-based inactivated influenza vaccine during the 2017-18 influenza season. PLoS ONE.

[31] Foppa IM, Haber M, Ferdinands JM, Shay DK (2013). The case test-negative design for studies of the effectiveness of influenza vaccine. Vaccine.

[32] Jackson ML, Nelson JC (2013). The test-negative design for estimating influenza vaccine effectiveness. Vaccine.

[33] Ainslie KEC, Shi M, Haber M, Orenstein WA (2017). On the bias of estimates of influenza vaccine effectiveness from test-negative studies. Vaccine.

[34] Gordon N, Lin T (2016). The Kaiser Permanente Northern California adult member health survey. Perm. J..

[35] Messer LC (2006). The development of a standardized neighborhood deprivation index. J. Urban Health.

[36] Zerbo O (2020). Individual and neighborhood factors associated with failure to vaccinate against influenza during pregnancy. Am. J. Epidemiol..

