# Supplementary material for: Maternal SARS-CoV-2 vaccination and infant protection against SARS-CoV-2 during the first six months of life
Source: Nat Commun. 2023 Feb 28;14:894. doi: 10.1038/s41467-023-36547-4 (PMC9974935; doi:10.1038/s41467-023-36547-4)
Supplement: Supplementary file 1 — Supplementary Information [file 41467_2023_36547_MOESM1_ESM.pdf]

Supplemental Table 1. Effectiveness of COVID-19 Vaccination During Pregnancy Against Infant SARS-CoV-2 Infection: Cohort Design, unadjusted for patient covariates

	**First 2 months of life**			**First 4 months of life**			**First 6 months of life**		
Infant observation period by predominant SARS-CoV-2 variant, and mother's vaccination status	Positive test N	Crude incidence rate[1]	Crude[2] VE (95% CI)	Positive test N	Crude incidence rate[1]	Crude[2] VE (95% CI)	Positive test N	Crude incidence rate[1]	Crude[2] VE (95% CI)
Delta period									
Unvaccinated during pregnancy	54	5.47	Reference	118	5.10	Reference	190	4.78	Reference
Received ≥2 doses during pregnancy	8	0.75	86 (71, 93)	27	1.43	72 (57, 81)	37	1.56	66 (52, 76)
Received only 1 dose during pregnancy*	0	0.00	no estimate	0	0.00	no estimate	4	1.33	72 (24, 89)
Omicron period									
Unvaccinated during pregnancy	54	16.98	Reference	140	18.32	Reference	302	21.11	Reference
Received ≥2 doses during pregnancy	54	16.30	29 (-4, 51)	175	17.25	21 (1, 37)	320	16.34	22 (9, 33)
Received only 1 dose during pregnancy*	1	7.87	51 (-72, 93)	8	17.79	24 (-34, 63)	23	22.73	-1 (-34, 33)

[1] Rate per 100 person-years

[2] Crude VE is adjusted for calendar time only.

* Most 1 dose only vaccines were received during the third trimester

VE=Vaccine effectiveness

Calendar day was used as the underlying scale to ensure that infants of vaccinated and unvaccinated mothers are compared on the same calendar days.

Supplemental Table 2. Effectiveness of COVID-19 Vaccination During Pregnancy Against Infant SARS-CoV-2 Infection by Infant Age at Testing and by Virus Variant: Test Negative Design

	First 2 months of life			First 4 months of life			First 6 months of life		
Infant observation period by predominant SARS-CoV-2 variant, and mother's vaccination status	Positive test N	Negative test N	Adjusted[2] VE (95% CI)	Positive test N	Negative test N	Adjusted[2] VE (95% CI)	Positive test N	Negative test N	Adjusted[2] VE (95% CI)
Delta period									
Unvaccinated during pregnancy	54	646	Reference	118	1193	Reference	190	2101	Reference
Received ≥2 doses during pregnancy	8	614	95 (76, 99)	27	1056	70 (52, 82)	37	1405	61 (42, 74)
Received only 1 dose during pregnancy[*]	0	60	no estimate	0	109	no estimate	4	217	76 (31, 92)
Omicron period									
Unvaccinated during pregnancy	54	273	Reference	140	464	Reference	302	792	Reference
Received ≥2 doses during pregnancy	54	328	43 (-4, 69)	175	714	36 (11, 55)	320	1250	41 (25, 53)
Received only 1 dose during pregnancy[*]	1	10	-27 (-91, 84)	8	30	36 (-50, 80)	23	63	16 (-35, 55)

[1] Rate per 100 person-years
[2] Adjusted for mother's age, race/ethnicity, NDI quartile, insurance payor, KPNC facility, pre-pregnancy BMI, diabetes, hypertension, parity, and child age.
[*]Most 1 dose only vaccines were received during the third trimester
VE= Vaccine effectiveness.

Supplemental Table 3. Effectiveness of COVID-19 Vaccination During Pregnancy Against Infant SARS-CoV-2 Infection by Infant Age at Testing and by Virus Variant: Test Negative Design, unadjusted for any patient covariates

	**First 2 months of life**			**First 4 months of life**			**First 6 months of life**		
Infant observation period by predominant SARS-CoV-2 variant, and mother's vaccination status	Positive test N	Negative test N	Crude[2] VE (95% CI)	Positive test N	Negative test N	Crude[2] VE (95% CI)	Positive test N	Negative test N	Crude[2] VE (95% CI)
Delta period									
Unvaccinated during pregnancy	54	646	Reference	118	1193	Reference	190	2101	Reference
Received ≥2 doses during pregnancy	8	614	86 (70, 93)	27	1056	73 (59, 83)	37	1405	68 (54, 78)
Received only 1 dose during pregnancy*	0	60	no estimate	0	109	no estimate	4	217	80 (46, 93)
Omicron period									
Unvaccinated during pregnancy	54	273	Reference	140	464	Reference	302	792	Reference
Received ≥2 doses during pregnancy	54	328	24 (-16, 51)	175	714	24 (-1, 42)	320	1250	33 (25, 40)
Received only 1 dose during pregnancy*	1	10	-12 (-94, 92)	8	30	7 (-55, 61)	23	63	22 (-1, 41)

[1] Rate per 100 person-years
[2] Unadjusted for patient covariates.
* Most 1 dose only vaccines were received during the third trimester
VE=Vaccine effectiveness

Supplemental Table 4. Effectiveness of COVID-19 Vaccination Before and During Pregnancy Against Infant SARS-CoV-2 Infection. Analysis Includes Only Infants Whose Mothers had Some Vaccine Doses Prior to Pregnancy onset and Some During Pregnancy: Cohort Design

	**First 2 months of life**			**First 4 months of life**			**First 6 months of life**		
Infant observation period by predominant SARS-CoV-2 variant, and mother's vaccination status	Positive test N	Crude incidence rate[1]	Adjusted[2] VE (95% CI)	Positive test N	Crude incidence rate[1]	Adjusted[2] VE (95% CI)	Positive test N	Crude incidence rate[1]	Adjusted[2] VE (95% CI)
Omicron period									
Unvaccinated before and during pregnancy	54	16.98	Reference	140	18.32	Reference	302	21.11	Reference
1 dose before, 1 dose during pregnancy	6	9.86	46 (-23, 77)	18	12.59	16 (-28, 50)	26	12.95	3 (-32, 36)
1 dose before, 2+ doses during pregnancy	1	1.59	89 (25, 98)	4	3.14	73 (29, 90)	9	5.74	48 (-1, 74)
2 doses before, 1 dose during pregnancy	29	5.34	45 (1, 69)	54	6.45	30 (-4, 53)	62	6.79	23 (-8, 45)

[1] Rate per 100 person-years

[2] Adjusted for mother's age, race/ethnicity, NDI quartile, insurance payor, KPNC facility, pre-pregnancy BMI, diabetes, hypertension, parity, and child age.

VE= Vaccine effectiveness.

Calendar day was used as the underlying scale to ensure that infants of vaccinated and unvaccinated mothers are compared on the same calendar days.
