# Supplementary material for: Maternal SARS-CoV-2 vaccination and infant protection against SARS-CoV-2 during the first six months of life
Source: Nat Commun. 2023 Feb 28;14:894. doi: 10.1038/s41467-023-36547-4 (PMC9974935; doi:10.1038/s41467-023-36547-4)
Supplement: Supplementary file 2 — Reporting Summary [file 41467_2023_36547_MOESM2_ESM.pdf]

nature portfolio

Corresponding author(s): Ousseny Zerbo

Last updated by author(s): Jan 26, 2023

Reporting Summary

Nature Portfolio wishes to improve the reproducibility of the work that we publish. This form provides structure for consistency and transparency in reporting. For further information on Nature Portfolio policies, see our Editorial Policies and the Editorial Policy Checklist.

Statistics

For all statistical analyses, confirm that the following items are present in the figure legend, table legend, main text, or Methods section.

n/a	Confirmed	
☐	☒	The exact sample size (*n*) for each experimental group/condition, given as a discrete number and unit of measurement
☒	☐	A statement on whether measurements were taken from distinct samples or whether the same sample was measured repeatedly
☐	☒	The statistical test(s) used AND whether they are one- or two-sided<br>*Only common tests should be described solely by name; describe more complex techniques in the Methods section.*
☐	☒	A description of all covariates tested
☒	☐	A description of any assumptions or corrections, such as tests of normality and adjustment for multiple comparisons
☐	☒	A full description of the statistical parameters including central tendency (e.g. means) or other basic estimates (e.g. regression coefficient) AND variation (e.g. standard deviation) or associated estimates of uncertainty (e.g. confidence intervals)
☒	☐	For null hypothesis testing, the test statistic (e.g. *F*, *t*, *r*) with confidence intervals, effect sizes, degrees of freedom and *P* value noted<br>*Give P values as exact values whenever suitable.*
☒	☐	For Bayesian analysis, information on the choice of priors and Markov chain Monte Carlo settings
☒	☐	For hierarchical and complex designs, identification of the appropriate level for tests and full reporting of outcomes
☒	☐	Estimates of effect sizes (e.g. Cohen's *d*, Pearson's *r*), indicating how they were calculated

Our web collection on statistics for biologists contains articles on many of the points above.

Software and code

Policy information about availability of computer code

Data collection	We used electronic health records data captured through Kaiser Permanente HealthConnect®, a customized EPIC system February 2020 version.
Data analysis	All analyses were conducted using SAS software, v9.4

For manuscripts utilizing custom algorithms or software that are central to the research but not yet described in published literature, software must be made available to editors and reviewers. We strongly encourage code deposition in a community repository (e.g. GitHub). See the Nature Portfolio guidelines for submitting code & software for further information.

Data

Policy information about availability of data

All manuscripts must include a data availability statement. This statement should provide the following information, where applicable:

- Accession codes, unique identifiers, or web links for publicly available datasets
- A description of any restrictions on data availability
- For clinical datasets or third party data, please ensure that the statement adheres to our policy

The data cannot be shared publicly because the data contain potentially identifying or sensitive patient information and is legally restricted by Kaiser Permanente Northern California. Data are available for researchers who meet the criteria for access to Kaiser Permanente Northern California confidential data. Data requests may be sent to Kaiser Permanente Division of Research: DOR.IRB.Submissions@kp.org.

Human research participants

Policy information about studies involving human research participants and Sex and Gender in Research.

Reporting on sex and gender	In this study the exposure was only assessed among pregnant persons. For the outcomes, we did not consider any sex differences.
Population characteristics	Please see above
Recruitment	The study used electronic health records; no recruitment was conducted.
Ethics oversight	The Kaiser Permanente Northern California Institutional review board approved and waived consent for this study.

Note that full information on the approval of the study protocol must also be provided in the manuscript.

Field-specific reporting

Please select the one below that is the best fit for your research. If you are not sure, read the appropriate sections before making your selection.

☐ Life sciences ☒ Behavioural & social sciences ☐ Ecological, evolutionary & environmental sciences

For a reference copy of the document with all sections, see nature.com/documents/nr-reporting-summary-flat.pdf

Behavioural & social sciences study design

All studies must disclose on these points even when the disclosure is negative.

Study description	This is a retrospective cohort study based on patient electronic health record data. The method is purely quantitative.
Research sample	The study was conducted among a cohort of infants ages 0 - 6 months, born between December 15, 2020, and May 31, 2022 at Kaiser Permanente Northern California (KPNC), an integrated healthcare delivery organization that provides comprehensive health care to approximately 4.4 million members. KPNC members are similar to the broad catchment population in Northern California in terms of sociodemographic characteristic.<br>The final study population included 30311 infants who were KPNC members at least 2 months after birth. The mean age at pregnancy onset was 31.62 years (standard deviation 4.66 years). Most mothers (66.14%) were between ages 25 and <35 years, and more than a quarter (27.27%) were of Asian race, 5.16% were Black, 24.44% were of Hispanic ethnicity and 37.57% were White.<br>This sample was included because it has complete information on maternal vaccination status, complete follow up from pregnancy onset to delivery and had information on infants.
Sampling strategy	All infants who met inclusion criteria were included without any sampling. We did not conduct any sample size calculation because the goal was to include all eligible infants.
Data collection	No dedicated data collection was performed. The entire study is based on existing electronic health record data collected as part of clinical care. The data analyst used an inclusion and exclusion criteria listed in Figure 1 of the manuscript to create the analytical files. The data analyst was not blinded with regard to study hypothesis. The analytical sample was created using a set of SAS codes.
Timing	The study was conducted among a cohort of infants born between December 15, 2020, and May 31, 2022.
Data exclusions	From the initial cohort, the study excluded the following infants born to: 1) mothers who were not between ages 16 and 50 years at pregnancy onset; 2) mothers who did not have a primary KPNC facility assignment; 3) mothers who were not continuous KPNC members from December 15, 2020 until delivery 4) mothers who had a positive nasal/throat swab for SARS-CoV-2 by polymerase chain reaction (PCR) prior to pregnancy onset; 5) mothers who had a positive SARS-CoV-2 antibody test documented by KPNC prior to onset of pregnancy; 6) mothers who received one or more doses of COVID-19 vaccine prior to pregnancy onset. We excluded these infants because we were primarily interested in estimating the effectiveness of at least 2 doses of mRNA vaccines received during pregnancy; 7) mothers who received other COVID-19 vaccine than mRNA vaccine during pregnancy; 8) mothers who did not receive their mRNA vaccinations in accordance with CDC recommendations – e.g., the timing between dose 1 and dose 2 was not within the recommended intervals; and 9) infants who did not become KPNC members within two calendar months of their birth. No other exclusion criteria were applied.<br>We excluded 21891 (35.2%) based on maternal exclusion criteria and 10412 (16.8%) after applying infant exclusion criteria (Figure 1)
Non-participation	Between December 15, 2020, and May 31,2022, we identified 62,117 infants born at Kaiser Permanente Northern California. from this cohort, we excluded 31,829 infants who did not meet all inclusion criteria. The final cohort included 30,288 infants. No participants were involved in the study because it was based on data already collected.
Randomization	No randomization was performed because this is an observational study.

Reporting for specific materials, systems and methods

We require information from authors about some types of materials, experimental systems and methods used in many studies. Here, indicate whether each material, system or method listed is relevant to your study. If you are not sure if a list item applies to your research, read the appropriate section before selecting a response.

Materials & experimental systems

n/a	Involved in the study
☒	☐ Antibodies
☒	☐ Eukaryotic cell lines
☒	☐ Palaeontology and archaeology
☒	☐ Animals and other organisms
☒	☐ Clinical data
☒	☐ Dual use research of concern

Methods

n/a	Involved in the study
☒	☐ ChIP-seq
☒	☐ Flow cytometry
☒	☐ MRI-based neuroimaging
